# Supervised Learning in SNN via Reward-Modulated Spike-Timing-Dependent Plasticity for a Target Reaching Vehicle

**DOI:** 10.3389/fnbot.2019.00018

**Published:** 2019-05-03

**Authors:** Zhenshan Bing, Ivan Baumann, Zhuangyi Jiang, Kai Huang, Caixia Cai, Alois Knoll

**Affiliations:** ^1^Chair of Robotics, Artificial Intelligence and Embedded Systems, Department of Informatics, Technical University of Munich, Munich, Germany; ^2^Department of Data and Computer Science, Sun Yat-Sen University, Guangzhou, China; ^3^Peng Cheng Laboratory, Shenzhen, China

**Keywords:** spiking neural network, R-STDP, supervised learning, end-to-end control, autonomous locomotion

## Abstract

Spiking neural networks (SNNs) offer many advantages over traditional artificial neural networks (ANNs) such as biological plausibility, fast information processing, and energy efficiency. Although SNNs have been used to solve a variety of control tasks using the Spike-Timing-Dependent Plasticity (STDP) learning rule, existing solutions usually involve hard-coded network architectures solving specific tasks rather than solving different kinds of tasks generally. This results in neglecting one of the biggest advantages of ANNs, i.e., being general-purpose and easy-to-use due to their simple network architecture, which usually consists of an input layer, one or multiple hidden layers and an output layer. This paper addresses the problem by introducing an end-to-end learning approach of spiking neural networks constructed with one hidden layer and reward-modulated Spike-Timing-Dependent Plasticity (R-STDP) synapses in an all-to-all fashion. We use the supervised reward-modulated Spike-Timing-Dependent-Plasticity learning rule to train two different SNN-based sub-controllers to replicate a desired obstacle avoiding and goal approaching behavior, provided by pre-generated datasets. Together they make up a target-reaching controller, which is used to control a simulated mobile robot to reach a target area while avoiding obstacles in its path. We demonstrate the performance and effectiveness of our trained SNNs to achieve target reaching tasks in different unknown scenarios.

## 1. Introduction

Despite the success of traditional artificial neural networks (ANNs) in learning complex non-linear functions, the interest of spiking neural networks (SNNs) is steadily increasing due to the fact that SNNs offer many fundamental and inherent advantages over traditional ANNs, such as biological plausibility (Maass, 1997), rapid information processing (Thorpe et al., 2001; Wysoski et al., 2010), and energy efficiency (Drubach, 2000; Cassidy et al., 2014).

Since traditional ANN computing units process signals in the form of continuous activation functions, they can be interpreted as their average pulse frequencies over a time window. Different from this, SNNs process information in the form of pulses or spikes, which is much more similar to the natural nervous system, and therefore, more biologically realistic and plausible. An advantage of this form of information processing is the possibility of not only encoding spatial information like traditional ANNs do, but also adding temporal information in the form of the precise timing of spikes (Maass, 1997). This eliminates the need for an averaging time window and allows for processing information in continuous time, greatly reducing response latencies (Frémaux et al., 2013). Since SNNs are able to transmit and receive large volumes of data encoded by the relative timing of only a few spikes, this also leads to the possibility of very fast and energy-efficient computing. For example, experiments have demonstrated that the visual pattern analysis and pattern classification can be carried out by humans in just 100 ms, in spite of the fact that it involves a minimum of 10 synaptic stages from the retina to the temporal lobe (Thorpe et al., 2001). Furthermore, in terms of energy efficiency, maintaining the sufficient functions of the nervous system to perform various tasks requires a continuous energy supply (Schoettle and Sivak, 2014). Yet, the human brain only needs remarkably low power consumption, which is around 20 W of power (Drubach, 2000). Overall, SNNs have great potential to offer an accurate and efficient way to model the principles underlying neural structures devoted to locomotion control in living creatures. Hence, mobile robots will be able to manage their weaknesses of carrying limited computing resources and power supply based on SNN-based controllers.

However, training these kinds of networks is notoriously difficult, since the error back-propagation mechanism commonly used in conventional neural networks cannot be directly transferred to SNNs due to the non-differentiability at spike times. Although there are successful attempts to combine the advantages of SNNs and the back-propagation mechanism together (Esser et al., 2015; Neftci et al., 2017), they basically transfer the continuous errors into rate-based or probability-based spikes, which gave away some of the inherent advantages of SNNs, such as the temporal information encoded by the timing of spikes.

Although a variety of learning rules for SNNs have been proposed in the past (Ponulak and Kasinski, 2011; Bing et al., 2018), solving different tasks usually involves constructing a specific network architecture suited to solve them, limited by lack of efficient and versatile training solutions. Initially, SNN-based control tasks were performed by manually setting network weights (e.g., Indiveri, 1999; Lewis et al., 2000; Ambrosano et al., 2016). Although this approach is able to solve simple behavioral tasks, such as wall following (Wang et al., 2009) or lane keeping (Kaiser et al., 2016), it is only feasible for lightweight networks with few connections or simple network architectures without hidden layers.

Meanwhile, many other indirect and direct training methods for SNNs have been proposed and demonstrated on robotic applications. For direct training methods, researchers have been working constantly on studying the underlying mechanism in brains. As one of the fundamental rules for synaptic activities, the functionality of the spike-timing-dependent plasticity (STDP) has revealed that one synaptic connection is affected by the precise timing of pre- and post-synaptic spikes. On this basis, the STDP learning rule is used in robotic control. Neuroscience studies also reveal that the brain modifies the outcome of STDP synapses using one or more chemicals emitted by given neurons. This mechanism inspires a new method for training SNNs and is known as reward-modulated spike-timing-dependent plasticity (R-STDP) (Izhikevich, 2007). Since the R-STDP can modulate an SNN with external signals that are sparse or delayed, this method is well suited for mobile robotic tasks.

Although the R-STDP learning rule assembles the natural learning process better, there still exist many problems for widespread implementations. The challenges for implementing R-STDP on mobile robotic applications lie in several aspects. First, there has been lacking of a unified learning paradigm that can be easily applied to different tasks and assign neuron modulations regardless of the multi-layered SNN structure. Second, in order to shape desired behaviors of the robot, defining proper rewards is important but complicated. Besides, the working mechanism of the reward-based neuron modulator is still unclear in multi-layered SNNs. Third, the R-STDP learning rule, similar to reinforcement learning, requires the agents to explore and interact with the environment randomly at the beginning. Improper parameters will consume long period of time or even fail the tasks with high probability.

On the other hand, indirect training methods for SNNs based on biological synaptic plasticity offers a fast and feasible way to construct a robotic controller. The reasons are two-fold. First, pre-acquired knowledge from traditional control methods can quickly shape the behaviors of a robot and offer a basic dataset to train the network based on the supervised learning framework (Bouganis and Shanahan, 2010). Second, the SNN with transferred policy can in return control the robot in an energy-efficient way, which can be achieved by running on a neuromorphic hardware (Blum et al., 2017).

To this end, our paper looks to explore an indirect SNN training approach based on the R-STDP learning rule and supervised learning framework.

A simple robot navigation task will be used as a case study to demonstrate the performance of our controller. Our main contributions are summarized as follows. First, a simulated target-reaching scenario is constructed and adapted with different traffic conditions for evaluating our proposed SNN-based controller, in which a Pioneer robot mounted with proximity sensors is regarded as the moving vehicle. This controller consists of a goal-approaching sub-controller, responsible for reaching a target area and an obstacle-avoiding sub-controller, responsible for avoiding obstacles in the robot's path. Second, a supervised R-STDP learning rule is proposed to train a simple one-hidden-layer SNN in an end-to-end learning fashion. The SNN-based controller computes the robot's proximity sensor readings and the direction of a target as inputs and the motor speed as the output. Finally, the training results of our target-reaching SNN are analyzed in terms of accuracy and then are further implemented in unknown scenarios to demonstrate the feasibility.

The rest of this paper is organized as follows. The related work is presented in section 2. Section 3 describes the modeling of the SNN and the supervised R-STDP learning rule. Section 4 describes the methods to generate the reference datasets. Section 5 introduces the architecture of the overall target-reaching controller and as well as its sub-controllers.

The training results and an evaluation of the performance of the different sub-controllers and the target-reaching controller as a whole in an unknown testing environment can be found in section 6. Finally, the work is summarized and future work is outlined in section 8. In the meantime, all of our codes and experiment demos can be found in the Supplementary Files.

## 2. Related Work

For many mobile robots, the ability to navigate in its environment is considered as the core function, which requires a robot to plan its path toward the goal location and avoid obstacles at the same time. In this study, performing navigation tasks on a mobile robot are used as a case study for evaluating our proposed SNN learning method.

Various model-based control methods for robotic navigation tasks have been widely investigated few decades ago (DeSouza and Kak, 2002; Kruse et al., 2013). For example, Brooks (1986) proposed a robust layered control architecture for mobile robots based on task-achieving behaviors. Bicho et al. (1998) presented an attractor dynamic approach to path planning, which only used low-level distance sensors to implement autonomous vehicle motion. Huang et al. (2006) proposed a steering potential function for vision-guided navigation tasks by using a single camera without recovering depth. Friudenberg and Koziol (2018) presented a new guidance method, which can allow a mobile robot interceptor to guide to, and rendezvous with, a moving target while avoiding obstacles in its path.

Meanwhile, the navigation behavior achieved by the biological intelligence in animal kingdoms exhibit excellent performance to avoid unpredictable obstacles agilely even in complex environments and outperform state-of-the-art robots in almost every aspects, such as agility, stability, and energy-efficiency.

In order to achieve similar outstanding performances, SNN architectures are increasingly being implemented for solving robotic navigation tasks using different training algorithms or running on neuromorphic hardware, due to those aforementioned advantages of SNNs.

Wang et al. (2008, 2014) constructed a single-layer SNN using a proximity sensor as the input and then trained it in tasks such as obstacle avoidance and target reaching. In this work, the propagation of the spikes through the network was precisely planned, such that the controlled robot car managed to avoid obstacles and long term plasticity was limited to only few synapses through STDP.

Beyeler et al. (2015) implemented a large-scale cortical neural network on a physical robot to achieve visual-guided navigation tasks, which produced similar trajectories as human behavioral data. However, most of the neurons in their network were still used as refined planar representations of the visual field by manually setting all the synaptic weights rather than training them. In the work of Cyr and Boukadoum (2012), where they used the classical conditioning to train a mobile robot to navigate through the environment, it was even stated that their architecture and initial synaptic weight matrix were intuitively hand-coded. In another example by Nichols et al. (2013), temporal difference learning was used to train a mobile robot in a self-organizing SNN for a wall-following task. However, each synaptic connection between neurons was formed when two specific neurons were active at the same time, which ultimately resulted in every single neuron in this multilayer structure having a specific predetermined function. Moeys et al. (2016) adopted the convolutional neural network (CNN) in the context of a predator/prey scenario. The events from an event-based vision sensor in each step are mapped into a frame of image based on the scene activity, which is fed into the CNN as the input. The network was off-line trained on labeled data and outputs simple left, right, or forward action directly. Milde et al. (2017) performed obstacle avoidance and target acquisition tasks with a robotic vehicle, on which an SNN takes event-based vision sensor as the input and runs on a neuromorphic hardware. It is worth mentioning that some fixed SNN architectures aim at solving a problem by imitating parts of structures of natural neural networks found in living organisms such as the withdrawal circuit of the Aplysia—a marine snail organism—in Alnajjar et al. (2008), olfactory learning observed in the fruit fly or honey bee in Helgadottir et al. (2013), or the cerebellum in Carrillo et al. (2008).

There are other SNN-based control approaches that are not necessarily dependent on the specific network architecture but with other drawbacks that limit their further utility. Bing et al. (2018) introduced an end-to-end learning approach of SNNs for a lane keeping vehicle. Their SNN was constructed with R-STDP synapses in an all-to-all connection and trained by the R-STDP learning rule. Even this end-to-end sensorimotor mapping drove the robot to follow lanes with different patterns, their network had a simple architecture only with the input layer and the output layer. Mahadevuni and Li (2017) solved goal approaching task by training an SNN using R-STDP. Shim and Li (2017) further proposed a multiplicative R-STDP by multiplying the current weight to the normal R-STDP and assigned the global award to all the synapses among two separated hidden layers in an SNN. In fact, most of the other approaches propose architectures that do not necessarily support hidden layers in their networks. In Vasilaki et al. (2009) and Frémaux et al. (2013), a map was fed into the network in the form of cells which were directly connected to the output layer neurons in a feed-forward and all-to-all manner. Each output neuron represented a different movement direction. In other approaches, such as Helgadottir et al. (2013) or Spüler et al. (2015), only a limited amount of synaptic connections employ synaptic plasticity while the majority of the synaptic strengths were fixed. Unfortunately, similar approaches only work for simple tasks rather than more complex tasks, which require precise tuning of many more degrees of freedom, e.g., one or more hidden layers, to solve the given task with satisfactory precision.

In summary, it can be seen that state-of-the-art SNNs based on R-STDP are still far from being general-purpose and easy-to-use, let alone the complexities in designing proper rewards or tunning a group of learning parameters. To remove the burden of designing complicated SNN architectures, indirect approaches for training SNNs are investigated. Foderaro et al. (2010) that induced changes in the synaptic efficacy through input spikes generated by a separate critic SNN. This external network was provided with control inputs as well as feedback signals and trained using a reward-based STDP learning rule. By minimizing the error between the control output and optimal control law, it was able to learn adaptive control of an aircraft. This was then used to train a simulated flying insect robot to follow a flight trajectory in Clawson et al. (2016). Similar ideas were presented by Zhang et al. (2012, 2013), Hu et al. (2014), and Mazumder et al. (2016) who trained a simple, virtual insect in a target reaching and obstacle avoidance task. However, this method is not suited for training an SNN on multi-dimensional inputs since the reward is dependent on the sign of the difference between the desired and actual SNN output. This also reveals another defect of most of current SNN-based control, which limits the use of SNN only to one-dimensional output.

To remove those aforementioned barriers, the architectures and learning rules used for SNNs should be able to operate on networks with hidden layer(s), multiple outputs, and continuous actions. These nice properties are also necessary in order for SNNs to extend and rival the concept of deep traditional ANNs using RL strategies or simply build the bridge between them. Therefore, we propose a novel SNN training approach based on R-STDP learning rule and the supervised learning framework. Based on this method, an SNN-based controller for mobile robot applications can be quickly and easily build with the help of traditional control knowledge.

## 3. Modeling of Spiking Neural Network

While most SNN architectures are specifically designed for the type of problems they are meant to solve, our SNN model together with the proposed learning rule aims at providing a simple universally usable network architecture similar to traditional ANNs that can be applied to a variety of problems, working in a black-box-like manner: The user should only think about in what form the inputs should be fed into the network and how the output is interpreted without having to worry about the specific way the neurons are connected.

### 3.1. Network Model

Our proposed network has a simple architecture consisting of an input layer encoding the sensory input vector with integrate-and-fire (IF) neurons, a hidden layer of leaky integrate-and-fire (LIF) neurons, and an output layer of LIF neurons, which provides the output spike trains that are decoded into an output vector. The network is a fully-connected feed-forward network, where the specific values of all parameters are listed in the Appendix.

The input neurons modeled can be interpreted as an IF neuron without leakage. Its firing threshold *v*_*th*_ is set as 1 mV and the neuron is modeled as

(1) $$\begin{array}{l} \frac{dv_{j}}{dt}=a\cdot x_{j}+b\text{.} \end{array}$$

**Figure 1:**
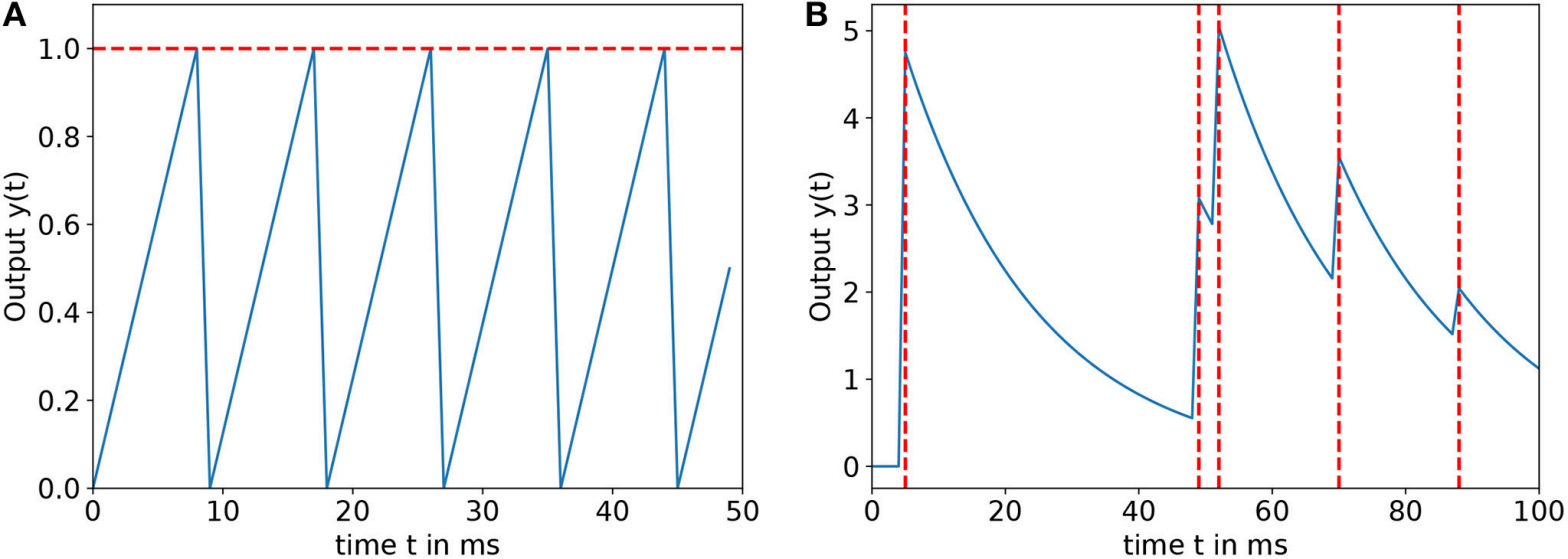
**(A)** Input encoding function for *a* = 0.2, *b* = 0.025, and *x*_*i*_ = 0.5 in a time window *T* = 50*ms*, the horizontal dashed line means the firing threshold. **(B)** Output function *y*(*t*) for α = 5, β = 0.05, and γ = 0 in a time window *T* = 100*ms*, the dashed vertical lines denote firing times of the output neuron.

*v*_*j*_ is the membrane potential of the neuron. *x* is the inject current. The parameter *b* is used to enable the input neuron firing even when there is no stimulus, since a spike will be generated every $\frac{1}{b}$ ms. This serves the purpose of helping to generate spikes for low input values in the time window *T* and thus enabling learning via STDP for inputs that would otherwise not have fired the input neurons in *T*. With the factor *a*, the build-up of the membrane potential can be scaled, limiting the amount of spikes generated in *T* for a maximum input to (*a* + *b*) × *T*. In this work, *a* is set to 0.2 and *b* = 0.025, resulting in the generation of one spike per time window for no input and 11 spikes for maximum input. An example is shown in Figure 1A.

The hidden and output layer consist of LIF neurons with thresholds *v*_*th,hidden*_ = 30 mV, *v*_*th,output*_ = 25 mV. The neurons in both layers share a refractory period τ_*ref*_ = 3 ms and their membrane time constant τ_*m*_ = 10 ms. The LIF neurons are modeled as follows:

(2) $$\begin{array}{l} \frac{dv_{j}\left(t\right)}{dt}=\left(-v_{j}\left(t\right)+PSP_{j}\left(t\right)\right)/\tau_{m}\text{.} \end{array}$$

Here, *v*_*j*_(*t*) is the *j*th neuron's membrane potential, *PSP*_*j*_(*t*) is the post-synaptic potential of the *j*th neuron. In this work, the *PSP* induced by a presynaptic spike has the shape of an alpha function, also referred to as alpha synapse (Gerstner and Kistler, 2002; Rothman and Silver, 2014).

The shape of the resulting PSP is approximated by a system of two differential equations:

(3) $$\begin{array}{l} \frac{dPSP_{j}(t)}{dt}=(-PSP_{j}(t)+i_{j}(t))/\tau_{s}\\ \frac{di_{j}(t)}{dt}=-i_{j}(t)/\tau_{s}+\sum_{t_{i}^{f}}w_{ij}\delta (t-t_{i}^{f})\text{,} \end{array}$$

where τ_*s*_ is a time constant controlling the decay of the *PSP*, *i*_*j*_(*t*) is the inject current, $t_{i}^{f}$ is the firing time of the *i*th neuron connecting to the neuron and δ(·) is the Dirac delta function. For the network to successfully learn a specific task, the exact shape of the *PSP* is not decisive. It simply has to be a function that allows for slow buildup of the membrane potential, resulting in firing the post-synaptic neuron earlier when the synaptic strength increases. Some mechanisms integrating the *PSP* by simply increasing the membrane potential at the arrival time of the pre-synaptic spike are not suited for this kind of network, since this would result in firing the post-synaptic neuron only at the same time the pre-synaptic neurons fires or after a certain delay has passed. And then, the STDP learning rule will not work with the delayed reward to adjust the synapses. The integration of the post-synaptic potential is chosen to be alpha-shaped, since the slow build up allows for integration of temporal information, which includes the timing of the post-synaptic spike and the pre-synaptic spike-timing and the strength of synaptic connection.

The output spike trains are decoded similarly to the leaky integrator equation in Clawson et al. (2016). To further increase the influence of the precise timing of the output spikes and reward early spiking, it was slightly changed to

(4) $$\begin{array}{l} y_{i}=\underset{t_{i}^{f}}{\sum }\alpha \frac{T-t_{i}^{f}}{T}\exp\left(\beta \left(t_{i}^{f}-t\right)\right)-\gamma , \end{array}$$

where α, β, γ are the output constants, *y*_*i*_ is the output of the *i*_*th*_ output neuron and $t_{i}^{f}$ are the firing times of this neuron. Figure 1B shows the development of the output function in the time window *T* for an exemplary spike train. All the neuron parameters are shown in Table 1.

**Table 1:** SNN parameters.

SNN simulation	Time window	*T* = 50*ms*
	Step size	*dt* = 1*ms*
Input layer	Input encoding	*a* = 0.2, *b* = 0.025
	Firing threshold	1
Hidden layer	Firing threshold	*v*_*th,hid*_ = 30*mV*
	Membrane time constant	τ_*m*_ = 10*ms*
	Synapse time constant	τ_*s*_ = 5*ms*
	Refractory period	τ_*ref*_ = 3*ms*
Output layer	Firing threshold	*v*_*th,out*_ = 30*mV*
	Membrane time constant	τ_*m*_ = 10*ms*
	Synapse time constant	τ_*s*_ = 5*ms*
	Refractory period	τ_*ref*_ = 3*ms*
	Output decoding	α = 20
		β = 0.1
		γ = 0

### 3.2. Supervised R-STDP Learning Rule

The basic idea underlying the proposed supervised R-STDP learning rule is the following: calculating the reward according to the supervised learning framework and strengthening a synaptic connection based on the combination effect of a dopamine reward and the STDP function, where STDP means strengthening a synaptic connection results in a faster buildup of the postsynaptic neuron potential when a presynaptic spike arrives, leading to the postsynaptic neuron firing earlier.

For this learning rule, the weight changes proposed by an STDP function are collected and a reward representing whether the output is higher or lower than the desired output is calculated after every simulation time window *T*. Then, this reward is used to change the synaptic connections of the network under the R-STDP learning rule.

The weights of the synaptic connections *w*_*ij*_, where *i* and *j* are the indices of the pre-synaptic and the post-synaptic neurons, respectively, are updated after the simulation time window *T* and follow the equations:

(5) $$\begin{array}{l} w_{ij}\left(t\right)=w_{ij}\left(t-\Delta t\right)+\Delta w_{ij}\left(t\right) \end{array}$$

(6) $$\begin{array}{l} \Delta w_{ij}\left(t\right)=\eta \times r_{ij}\left(t\right)\times STDP_{ij}\left(t\right)\times g_{ij}\left(t\right)\text{.} \end{array}$$

In this equation, *t* denotes the number of the current time window and Δ*t* = *T*. The learning rate η is a constant that regulates the learning speed of the SNN. Inspired by traditional ANNs, η starts at a maximum value η_*max*_ and decreases toward a minimum learning rate η_*min*_, such that the weight changes are becoming finer as the training progresses. It is updated after every training episode

(7) $$\begin{array}{l} \eta =\eta_{max}-\frac{\eta_{max}-\eta_{min}}{ep_{max}}\times ep_{curr}\text{,} \end{array}$$

where *ep*_*max*_ is the number of training episodes and *ep*_*curr*_ is the current training episode. The function *g*_*ij*_(*t*) is the synapses eligibility trace. As opposed to eligibility traces in reinforcement learning, here it models a phenomenon in biological neurons, where synapses with higher efficacies produce greater weight changes (Foderaro et al., 2010) and can be calculated as

(8) $$\begin{array}{l} g_{ij}\left(t\right)=1-c_{1}\times w_{ij}\times \exp\left(-c_{2}\times abs\left(w_{ij}\right)/w_{max}\right)\text{,} \end{array}$$

where *c*_1_ and *c*_2_ are positive constants. *c*_1_ is set to $\frac{1}{w_{max}}$ to make sure the eligibility traces only assume values between 1 and 0. The weight changes proposed by the STDP function are collected and represented by the term *STDP*_*ij*_. This mechanism is modeled with the help of two variables *a*_*ij,pre*_ and *a*_*ij,post*_ that are traces of the pre- and post-synaptic activity (Echeveste and Gros, 2015). They are governed by the following differential equations:

(9) $$\begin{array}{l} \tau_{pre}\frac{d\;a_{ij,pre}}{dt}=-a_{ij,pre}\\ \tau_{post}\frac{d\;a_{ij,post}}{dt}=-a_{ij,post} \end{array}$$

Upon occurrence of a pre-synaptic spike, *a*_*ij,pre*_ is updated and the proposed weight changes are modified:

(10) $$\begin{array}{l} a_{ij,pre}(t)=a_{ij,pre}(t-dt)+A_{pre}\\ STDP_{ij}(t)=STDP_{ij}(t-dt)+a_{ij,post}. \end{array}$$

When the post-synaptic neuron fires, *a*_*ij,post*_ is updated

(11) $$\begin{array}{l} a_{ij,post}(t)=a_{ij,post}(t-dt)-A_{post}\\ STDP_{ij}(t)=STDP_{ij}(t-dt)+a_{ij,pre}. \end{array}$$

The STDP function governed by these rules is equivalent to the STDP learning rule. The reason for using this function is to show the same behavior while being efficient and physiologically plausible as biological neurons, since they do not have a memory of all their fired spikes. The reward is represented by the term *r*_*ij*_. It can be seen as more of an adjustment than a reward, since it determines whether the SNN output has to be lowered or increased in order to reach the desired output.

After calculating the SNN's output *y*_*SNN,k*_, a reward variable that represents the relative deviation of each output from the desired value *y*_*con,k*_ (provided by the dataset) is calculated for each neuron indexed by *k*. As opposed to other R-STDP learning rules, there is no global reward signal, but every synapse is assigned its individual reward as

(12) $$\begin{array}{l} r_{k}=\left(\left|y_{con,k}|-|y_{SNN,k}\right|\right)/y_{max}\text{.} \end{array}$$

The value *y*_*max*_ is a maximum output that should not be exceeded. Thus, the synapse connecting the *j*th neuron in the hidden-layer and the *k*th neuron in the output layer are given a reward as

(13) $$\begin{array}{l} r_{jk}=r_{k}\text{.} \end{array}$$

To assign a reward to the synapses connecting the input to hidden neurons, it has to be calculated differently. In this paper, it is *backpropagated* through the layers: each hidden neuron has one synaptic connection to each output neuron, where the synapse is assigned a reward *r*_*k*_. With the help of the weights of those synaptic connections the reward of a hidden layer neuron (how much did it influence which output neuron) is calculated, following (14). This hidden layer neuron reward can now be assigned to the synapses connecting an input neuron to this neuron.

(14) $$\begin{array}{l} r_{i,j}=\left(\underset{k}{\sum }|w_{jk}|r_{k}\right)/\left(\underset{k}{\sum }|w_{jk}|\right) \end{array}$$

Here, *i* indexes the *i*th input layer neuron, *j* indexes the *j*th hidden layer neuron and *k* denotes the *k*th output layer neuron. With the proposed rule for setting rewards for synapses, an SNN construed with R-STDP synapses can be trained by the supervised learning framework with a dataset. Next, the user simply has to set the size of each layer of an SNN to achieve a desired behavior.

It should be noted that, while for the obstacle avoidance task, both output reward values for the hidden-output-synapses are calculated using (14), this was done slightly different for the goal approaching SNN. This is because the output of one neuron has to be precise, while the other neurons output simply has to be higher for our goal-approaching sub-controller to exhibit the aspired behavior. This will be explained in greater detail in section 5.2. For this rule, the most important part is to correctly judge whether an output has to be lowered or increased.

## 4. Reference Dataset

The target-reaching controller (TR) is supposed to drive the robot to reach a target area and avoid obstacles in its path. Each task is to be solved by a sub-SNN-controller trained by the supervised R-STDP learning rule, one for obstacle avoiding (OA) and one for goal approaching (GA).

Therefore, two datasets are created consisting of 500 input-output pairs, which are later used to train the sub-controllers to approximate their obstacle avoiding and goal approaching behaviors, respectively. The datasets are generated by simulating the locomotion tasks in the Virtual Robot Experimentation Platform (V-REP) (Rohmer et al., 2013).

### 4.1. Obstacle-Avoiding Dataset

The goal of the obstacle-avoiding sub-controller is to decide in which direction the mobile robot should take to avoid an encountered obstacle. For this purpose, an obstacle-avoiding reference controller based on simple if-then rules (Zadeh, 1992) is built. One piece of data in the dataset consists of the six sensor readings of the sensors *s*_1_–*s*_6_, the two output angles α_*OA,ref,L*_, α_*OA,ref,R*_ and the turn to take, i.e., left or right.

**Figure 2:**
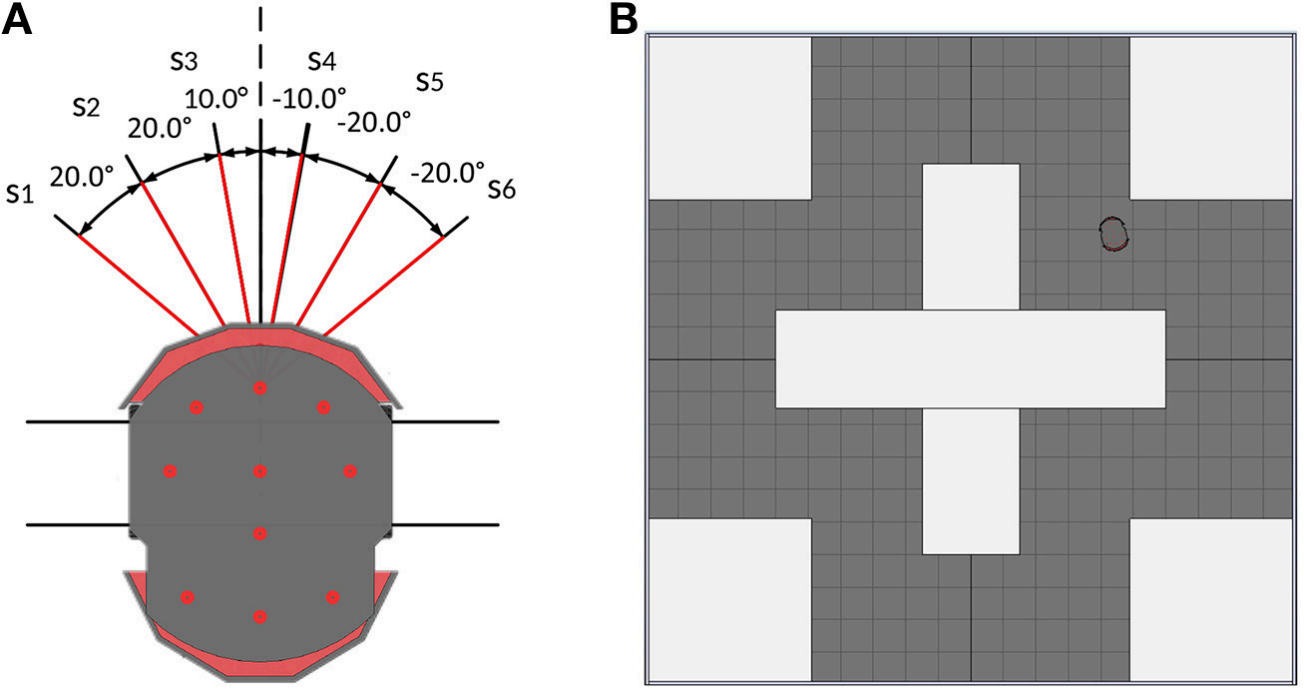
**(A)** The Pioneer robot with its 6 on-board sonar sensors. Red line denotes the sensors used for the obstacle-avoiding task. **(B)** Top-view of the V-REP scene that is used to collect the data for the obstacle-avoiding dataset.

The inputs of the obstacle-avoiding controller are the six central front sonar-sensors of the pioneer robot (see Figure 2A). The sensor reading *s*_*i*_ is given as the distance between the detected object to the *i*th sensor's position. Since sensors that do not detect anything return random values between 0 and 1 for the coordinates, an additional boolean variable *detect*_*i*_ is used for each sensor, which is *True* when an object is detected and *False* if not.

The outputs are two angles α_*OA,ref,L*_, α_*OA,ref,R*_, which are chosen based on the most central left and right sensors that do not detect any obstacle. Negative angles represent right turning, whereas positive angles represent left turning. To chose the turning angle α_*turn*_ for the robot, the angles are compared and the one with the lower absolute value is chosen. If two have the same absolute value, the sum of the left and right sensor readings are compared and the side with the lower overall sensor readings is chosen. The sensors are orientated at ±10, ±30, and ±50° at the front of the robot. To make sure the robot successfully avoids the detected obstacle, output angles are set to a value that is 10° higher than the respective sensor's orientation angles. If all sensors on one side detect an obstacle, the output angle is set to ±90°, implying a turn away from that obstacle. This turning rule is shown in **Algorithm 1**.

**Algorithm 1:**
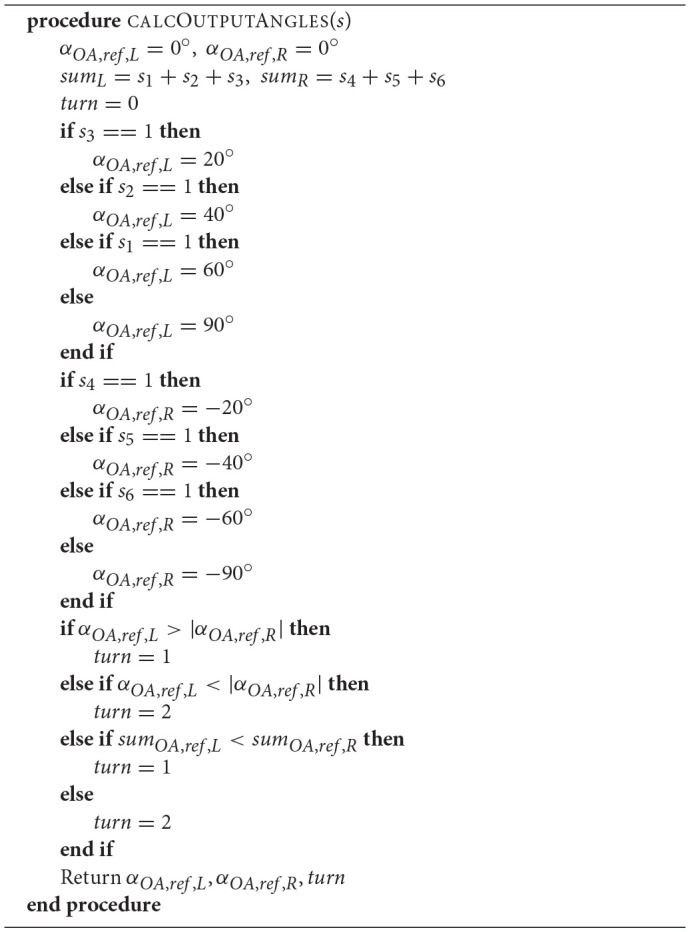
Algorithm to calculate the output angles for the reference obstacle avoiding controller

To create the dataset, the robot controlled by the reference controller drives around the training scene that can be seen in Figure 2B. Meanwhile, the robot saves the sensor readings as well as the outputs of **Algorithm 1** every 200 ms, if an obstacle is encountered. This scene is chosen as the training environment, because it ensures the robot takes both left and right turns while navigating through the scene. The controller then decides on the turn to take by choosing the angle with the smaller absolute value as the output angle α_*OA,ref*_. In the event that both output angles are the same, the angle calculation algorithm additionally returns the turn to take (1 for a left turn, 2 for a right turn) based on which side's detected obstacles are further away.

If all of the sensors *s*_1_–*s*_6_ return the following readings, no obstacle will be encountered, which means the robot will not collide with anything if moving forward.

(15) $$\begin{array}{l} \{\begin{array}{l} s_{1},\;s_{6}\geq 0.01\\ s_{2},\;s_{5}\geq 0.15\\ s_{3},\;s_{4}=1 \end{array} \end{array}$$

### 4.2. Goal-Approaching Dataset

**Figure 3:**
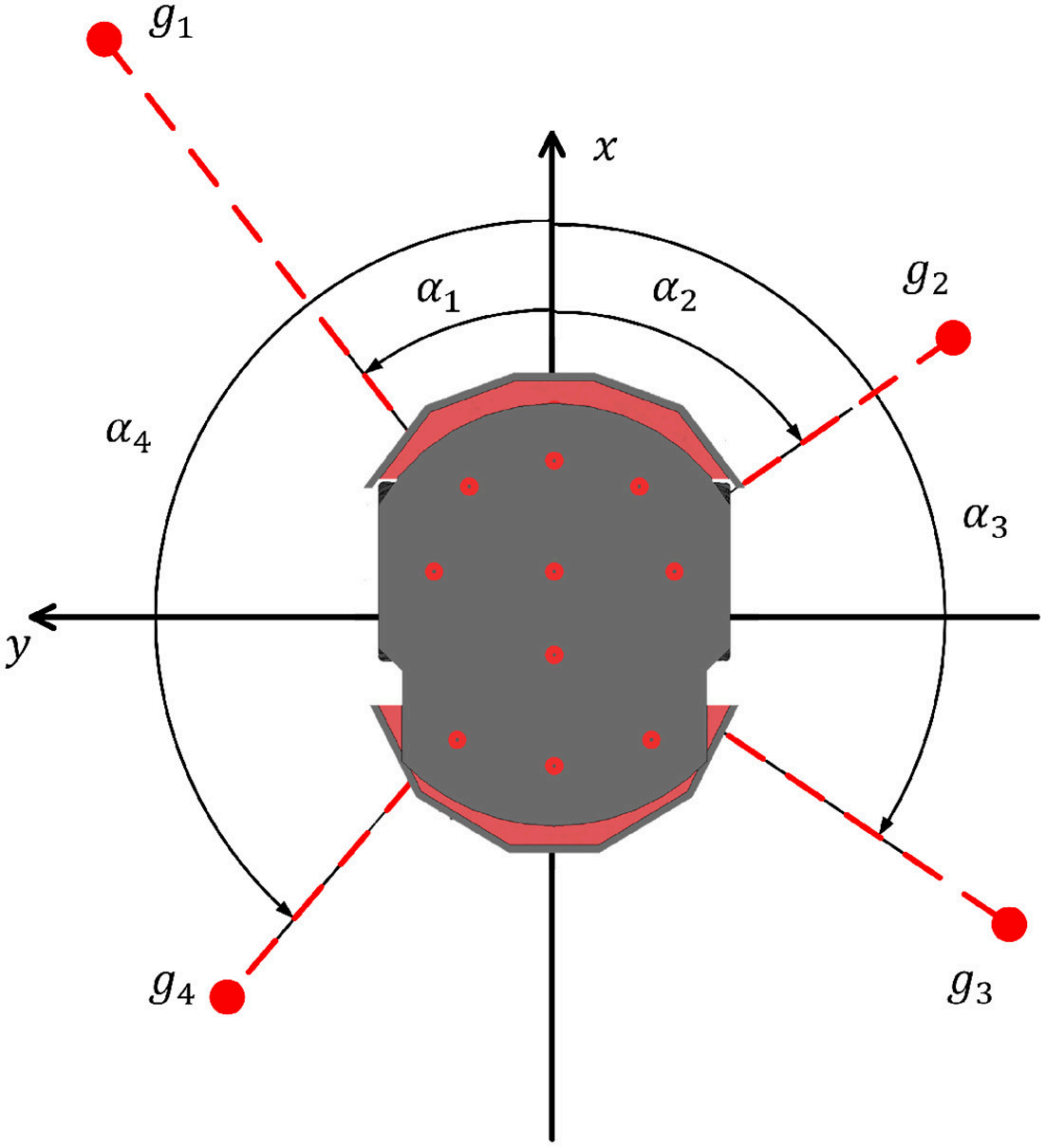
The Pioneer robot with different relative goal positions (*g*_1_ - *g*_4_) and the corresponding target angles (α_1_ - α_4_).

Another dataset is created to train the goal-approaching sub-controller in order to reach a pre-set target area. This controller gets the normalized vector $\vec{g}=\left(g_{x},g_{y}\right)$ from the Pioneer robot to the goal center as input and outputs a turning angle α_*GA,ref*_, which results in the robot to directly face the target. Figure 3 shows the Pioneer robot and its four imaginary goal positions *g*_1_–*g*_4_. It is easy to see that the controller should later calculate a left turning angle ($\alpha_{1}\;>\;0^{{}^{\circ}}$) for *y* > 0, a right angle ($\alpha_{2}\;<\;0^{{}^{\circ}}$) for *y* < 0, and an angle $|\alpha_{3/4}|\;>\;90^{{}^{\circ}}$ for *x* < 0. The controller's activity could be restrained due to being exposed to very high output angles (±180°) in training, while experiencing mostly low target angles when almost facing the goal. For this reason, all angles >90° are clipped at ±90°:

(16) $$\begin{array}{l} \alpha_{GA,ref}=\{\begin{array}{ll} arcsin(g_{y}) & \text{if }g_{x}>0;\\ 90^{{}^{\circ}} & \text{if }g_{y}>0,g_{x}<0;\\ -90^{{}^{\circ}} & \text{if }g_{y}<0,g_{x}<0. \end{array} \end{array}$$

To create the dataset, the coordinate pairs (*g*_*x*_, *g*_*y*_) are randomly generated and then normalized. The absolute value of the target angle is set to be >10°, because $\alpha_{GA,ref}\leq 10^{{}^{\circ}}$ is treated here as facing the target. From those normalized pairs (*g*_*x*_, *g*_*y*_), a target angle is calculated according to (16). Similar to the obstacle-avoiding SNN, the goal-approaching SNN later calculates two output angles, one for each side. For this reason, the angle of the side where the target is not located is set to ±180° to be consistent. One input-output pair then consists of the two parts of the goal vector *g*_*x*_, *g*_*y*_ and the two target angles α_*GA,ref,L*_, α_*GA,ref,R*_.

### 4.3. Calculating the Pioneer-Robot Motor Speeds

Since the reference datasets and SNN sub-controllers only provide the turning angles, it is necessary to translate them into actual motor speeds of the Pioneer robot in *rad*/*s*, where *v*_*forward*_ denotes the default motor speed when moving forward:

(17) $$\begin{array}{l} v_{left}=v_{forward}-\Delta v(\alpha )/2\\ v_{right}=v_{forward}+\Delta v(\alpha )/2. \end{array}$$

Δ*v*(α) is the difference in motor speeds necessary to achieve a turn of α degree in 1 s. The default forward speed is set to *v*_*forward*_ = 5.0*rad*/*s*.

## 5. Controller

In this section, the target-reaching (*TR*) control architecture is presented which consists of an obstacle-avoiding (*OA*) sub-controller and a goal-approaching (*GA*) sub-controller.

### 5.1. Target-Reaching Control Structure

**Figure 4:**
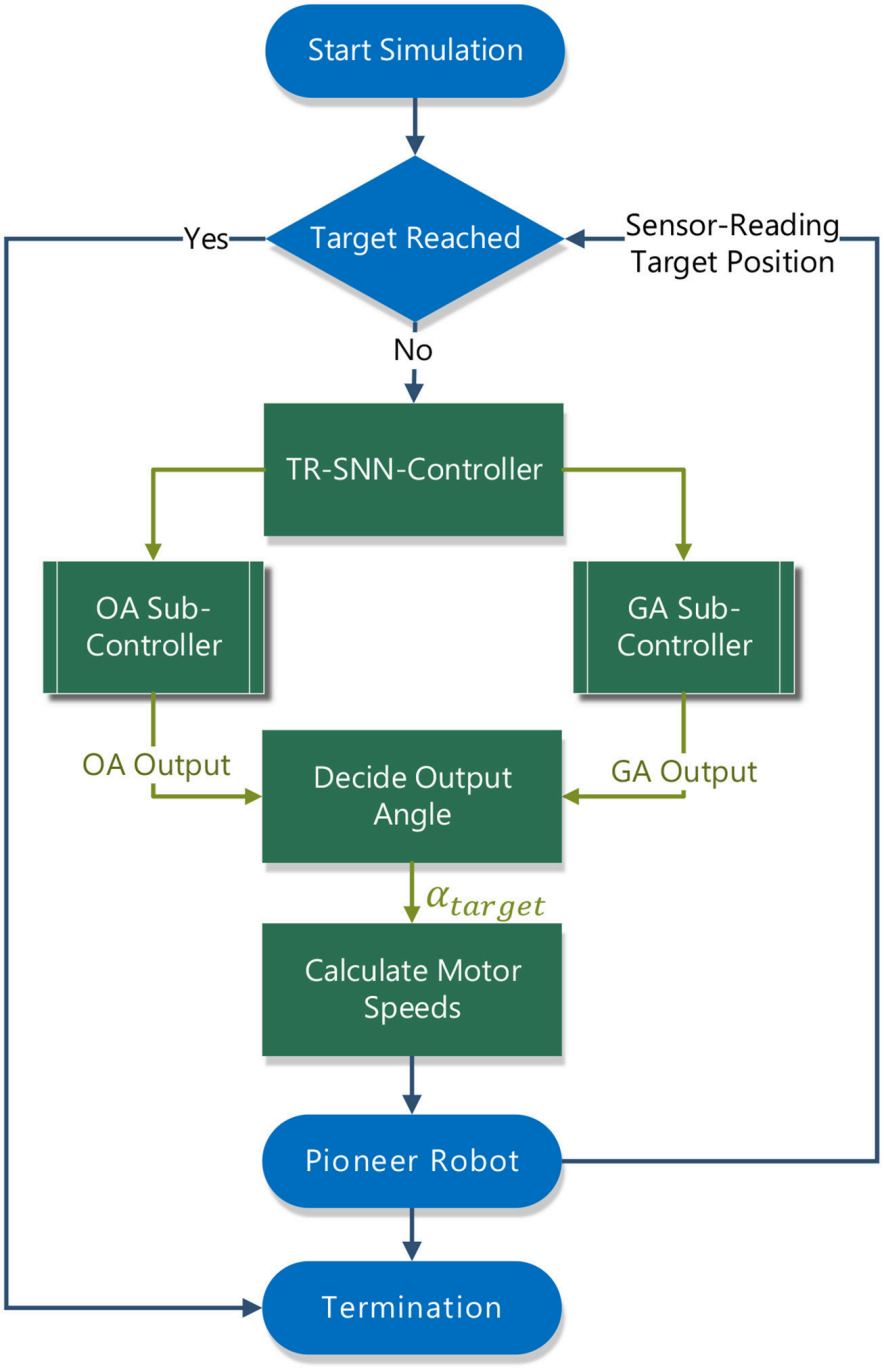
Structure of the Target-Reaching controller with its SNN sub-controllers communicating with the V-REP simulator.

Figure 4 shows the target-reaching control structure with which the Pioneer robot is controlled during simulation. Upon starting the simulation, V-REP passes the position of the target center *p*_*target*_ = (*p*_*target,x*_, *p*_*target,y*_) to the controller and the user is required to set the target area by specifying a radius *r*_*target*_. After every simulation time window, the controller is provided with the position of the Pioneer robot *p*_*p*3*dx*_ = (*p*_*p*3*dx,x*_, *p*_*p*3*dx,y*_), its proximity sensor readings *s*_1_–*s*_6_, and the normalized vector to the goal $\vec{g}$. It is then checked in every step whether the robot has reached the target area by calculating its distance *d* from the target center as:

(18) $$\begin{array}{l} d=\sqrt{{\left(p_{target,x}-p_{p3dx,x}\right)}^{2}+{\left(p_{target,y}-p_{p3dx,y}\right)}^{2}}\text{,} \end{array}$$

and then it is then compared to the initially specified target radius *r*_*target*_. If the robot is not in the target area, the SNN-based obstacle-avoiding sub-controller and the goal-approaching sub-controller will calculate their angle outputs to drive the robot for the next step, respectively. Finally, the motor speeds are further calculated according to the output angle of the controller.

The output layer of both sub-controllers is equipped with an additional neuron directly connected to the input layer, being referred to as obstacle neuron (*ON*) and target-facing neuron (*TN*) for the obstacle-avoiding and goal-approaching sub-controller in this work, respectively. These neurons are added to the architecture to judge whether the SNN sub-controllers should take action in controlling the mobile robot. The obstacle neuron checks whether a forthcoming obstacle has to be avoided. This is the case if the output *y*_*obst*_ of the obstacle neuron is higher than a threshold value *y*_*th,ON*_. Similarly, the robot is not facing the target if the target neuron output *y*_*target*_ < *y*_*th,TN*_. They are trained using the same learning rule as for the networks as a whole. With the output of these neurons, it is then decided which turning angle α_*turn*_ the robot has to take:

(19) $$\begin{array}{l} \alpha_{turn}=\{\begin{array}{ll} \alpha_{OA,L}, & if\;(y_{obst}>y_{th,ON})\;\wedge \;(\alpha_{OA,L}<|\alpha_{OA,R}|)\\ \alpha_{OA,R}, & if\;(y_{obst}>y_{th,ON})\;\wedge \;(\alpha_{OA,L}>|\alpha_{OA,R}|)\\ \alpha_{GA,L}, & if\;(y_{target}<y_{th,TN})\;\wedge \;(\alpha_{GA,L}<|\alpha_{GA,R}|)\\ \alpha_{GA,R}, & if\;(y_{target}<y_{th,TN})\;\wedge \;(\alpha_{GA,L}>|\alpha_{GA,R}|)\\ 0^{{}^{\circ}}, & else \end{array}\text{,} \end{array}$$

where α_*OA,L*_ and α_*OA,R*_ are the output of the obstacle-avoiding controller, α_*GA,L*_ and α_*GA,R*_ are the output of the goal-approaching controller. The subscript *L* and *R* represent turning left or right. To translate the output of each SNN sub-controllers into an angle, the following equation is used:

(20) $$\begin{array}{l} \alpha =\alpha_{min,OA/GA}+\left(\alpha_{max,OA/GA}-\alpha_{min,OA/GA}\right)\times \left(y_{SNN}/y_{max}\right)\text{,} \end{array}$$

where *y*_*SNN*_ and *y*_*max*_ are the output and the maximum output of each SNN, α_*min*_, and α_*max*_ are the range of the turning angle. This angle is then used to calculate the motor speeds of the Pioneer robot according to (17).

### 5.2. Goal-Approaching Sub-controller

**Figure 5:**
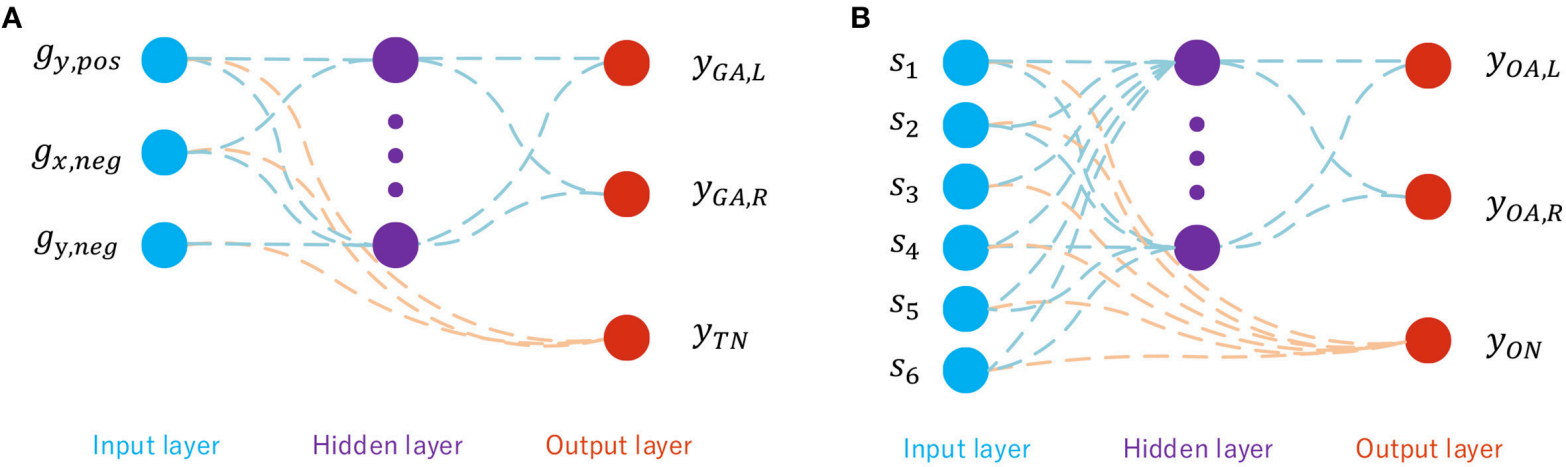
**(A)** Network topology of the GA sub-SNN. **(B)** Network topology of the OA sub-SNN.

Figure 5A shows the topology of the goal-approaching SNN with its three input neurons *g*_*y,pos*_, *g*_*x,neg*_, and *g*_*y,neg*_, which are the x- and y-components of the normalized goal vector $\vec{g}$ as shown in Figure 3. $\vec{g}$ consists of *g*_*x*_, *g*_*y*_ ∈ [−1, 1]. Since the inputs have to be real values between 0 and 1, they are split into a negative part *g*_*x,neg*_, *g*_*y,neg*_ and a positive part *g*_*x,pos*_ and *g*_*y,pos*_. Initially, both the negative and positive parts are set to zero. During the locomotion, if *g*_*y*_ is positive, its positive part is set to *g*_*y*_, otherwise the positive part is set to |*g*_*y*_|. The unset part remains zero. Since *g*_*x*_ and *g*_*y*_ stand in relation to each other as $g_{x}^{2}+g_{y}^{2}=1$, |*g*_*x*_| does not provide any additional information and therefore *g*_*x,pos*_ is not fed into the network. However, *g*_*x,neg*_ cannot be omitted, since *g*_*x,neg*_ < 0 implies that the output angle has to be >90°. As already mentioned in section 4.2, all output angles bigger than 90° are set to 90° in the dataset. For the hidden layer, there are 50 neurons inside.

The output neurons *y*_*GA,L*_ and *y*_*GA,R*_ are used for calculating the output angles α_*GA,L*_, α_*GA,R*_ according to (4) and (20). For goal approaching, α_*min,GA*_ is set to 10° and α_*max,GA*_ to 100°. *y*_*max*_ is the SNN's output for which α_*max,GA*_ is reached. For this problem, it is set to *y*_*max*_ = 5. The third output neuron is responsible for deciding whether the robot is facing the target or not, which is directly connected to the input neurons. The threshold *y*_*th,TN*_ is set to 5 in the simulation.

### 5.3. Obstacle Avoiding Sub-controller

The obstacle-avoiding sub-controller functions very similarly to the goal-approaching sub-controller. Its topology is shown in Figure 5B. It differs from the goal-approaching controller in two aspects, the first being the inputs fed into it. The input layer consists of six input neurons, each responsible for encoding one sensor reading. A sensor reading *s*_*i*_ is between 0 and 1, representing how many meters away the detected obstacle is located. Since a higher value represents a faraway obstacle and lower value for a close obstacle to the robot, $\bar{s_{i}}=1-s_{i}$ is fed into the network in order to ensure a network activity that is higher the closer an obstacle is. The output angles of the two motor neurons are as well calculated by (20) with $\alpha_{min,OA}=20^{{}^{\circ}}$, $\alpha_{max,OA}=100^{{}^{\circ}}$, and the same *y*_*max*_ = 5.

The neuron directly connected to the input layer evaluates whether an obstacle has to be avoided not; this is the case if its output is *y*_*obst*_ > 5.

## 6. Results and Discussion

We demonstrate the capabilities and efficiencies of our proposed end-to-end learning of spiking neural network based on supervised R-STDP by performing a target-reaching vehicle. Within V-REP, we first present our different scenarios for goal-approaching and obstacle-avoiding tasks. We then give and analyze the training performances of our SNN-based controllers in terms of training accuracy and errors. Finally, a group of overall target-reaching tasks are conducted in unknown scenarios to examine our proposed algorithms. The core concept of these tasks is to demonstrate a promising training method for SNN with a general-purpose and easy-to-use way.

### 6.1. Testing Environments

**Figure 6:**
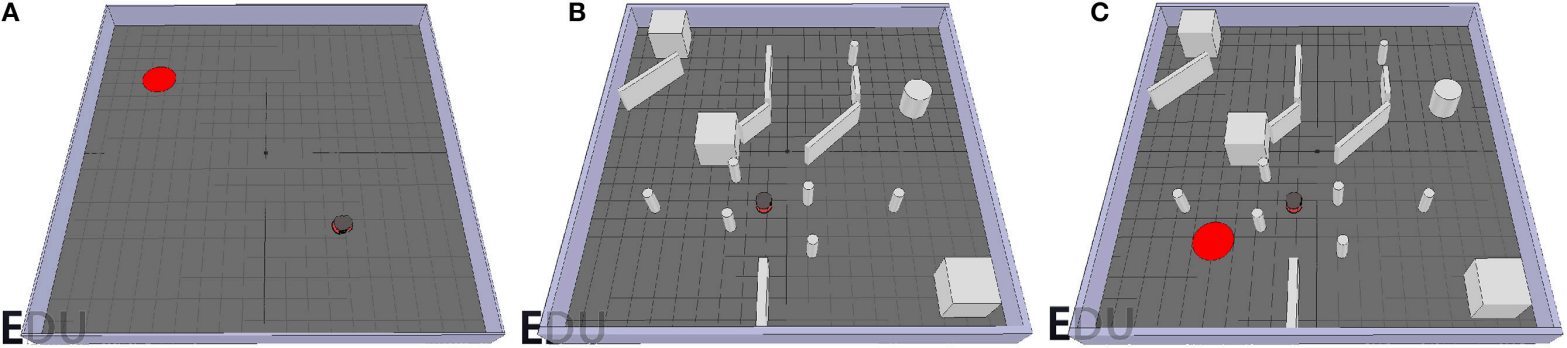
Simulation scenes used for testing the goal-approaching sub-controller. In each scenario, the red disc represents the goal position and the gray objects are the obstacles. **(A)**, the obstacle-avoiding sub-controller **(B)**, and the target-reaching controller **(C)**.

The environments used for testing the performance of the *GA* and *OA* controllers as well as the overall *TR* controller as a whole are presented in Figure 6. For the goal-approaching sub-controller, a target is represented by a red platform and placed in an open environment without obstacles (see Figure 6A). This allows for testing the ability of the robot to reach the target from different orientations without having to worry about colliding with an obstacle. For the obstacle-avoiding sub-controller, the mobile robot is driving around with the potential to collide with multiple obstacles, as shown in Figure 6B. Besides, the obstacles with different shapes and sizes (e.g., the thin pillars) have never been encountered before by the robot in the training scene (see Figure 2B). This is critical for verifying its ability to react correctly even to unknown stimulus. To test the performance of the target-reaching control structure as a whole, the target from the goal-approaching sub-controller scene is simply added in the obstacle-avoiding testing environment (Figure 6C).

The SNN models and the learning rule presented in section 3 are used to train the goal-approaching and obstacle-avoiding sub-controllers to mimic the output values for certain input vectors, both provided by their respective datasets. All controllers are trained for 100 episodes, where one episode consists of a set of 500 input-output pairs. The training accuracy denotes how often the sub-controllers chose the right turn (left or right), while the error represents the deviation from the desired output value.

### 6.2. Goal-Approaching Sub-controller

#### 6.2.1. Training Details

**Figure 7:**
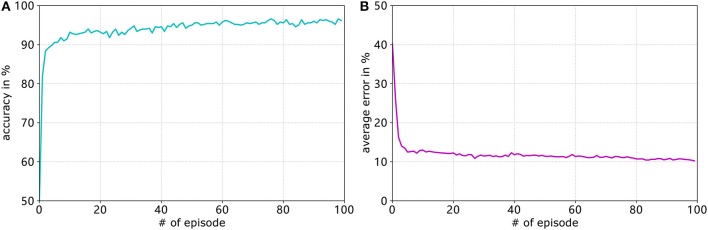
Development of the accuracy (blue graph in **A**) and average error (purple graph in **B**) of the goal-approaching sub-controller over the training episodes for *A*_+_ = 0.4, *A*_−_ = 0.42, η_*max*_ = 0.2, and η_*min*_ = 0.05.

Figure 7A shows the development of the accuracy and Figure 7B shows the average error of the goal-approaching sub-controller over the course of training. As can be seen, the accuracy quickly rises to a value of over 90% and then slowly keeps rising during the training process. The training terminates with a final accuracy of 96.2%. It should be noted that, after approximately 70 training episodes, the accuracy stagnates at values between 94.8 and 96.6%. Similar to the accuracy, the average error per episode falls to a value below 15% after only four training episodes and gradually reduces to an error of 10.24%. While it can be expected to continue to fall for more episodes, the error usually stagnates at a value of 10 ± 0.5%. The learning rates are set by trial and error for both sub-controllers. Generally speaking, the learning rates that are too low result in a much slower increase in accuracy, while the error rates even stagnate at values much worse than when choosing close-to-optimal learning rates. Meanwhile conversely, the accuracy and average error usually fluctuate before stagnating at a value far from the optimum.

The special neurons of each controller are trained separately by using the same learning rule to make their output approach the threshold value *y*_*th*_ for edge cases repeatedly until the average deviation falls under a certain level. For the target neuron (*TN*) the maximum deviation is set to 0.25 and the two edge cases are:

(21) $$\begin{array}{lll} g_{y,neg}=0.15, & g_{x,neg}=0, & g_{y,pos}=0;\\ g_{y,neg}=0, & g_{x,neg}=0, & g_{y,pos}=0.15. \end{array}$$

**Figure 8:**
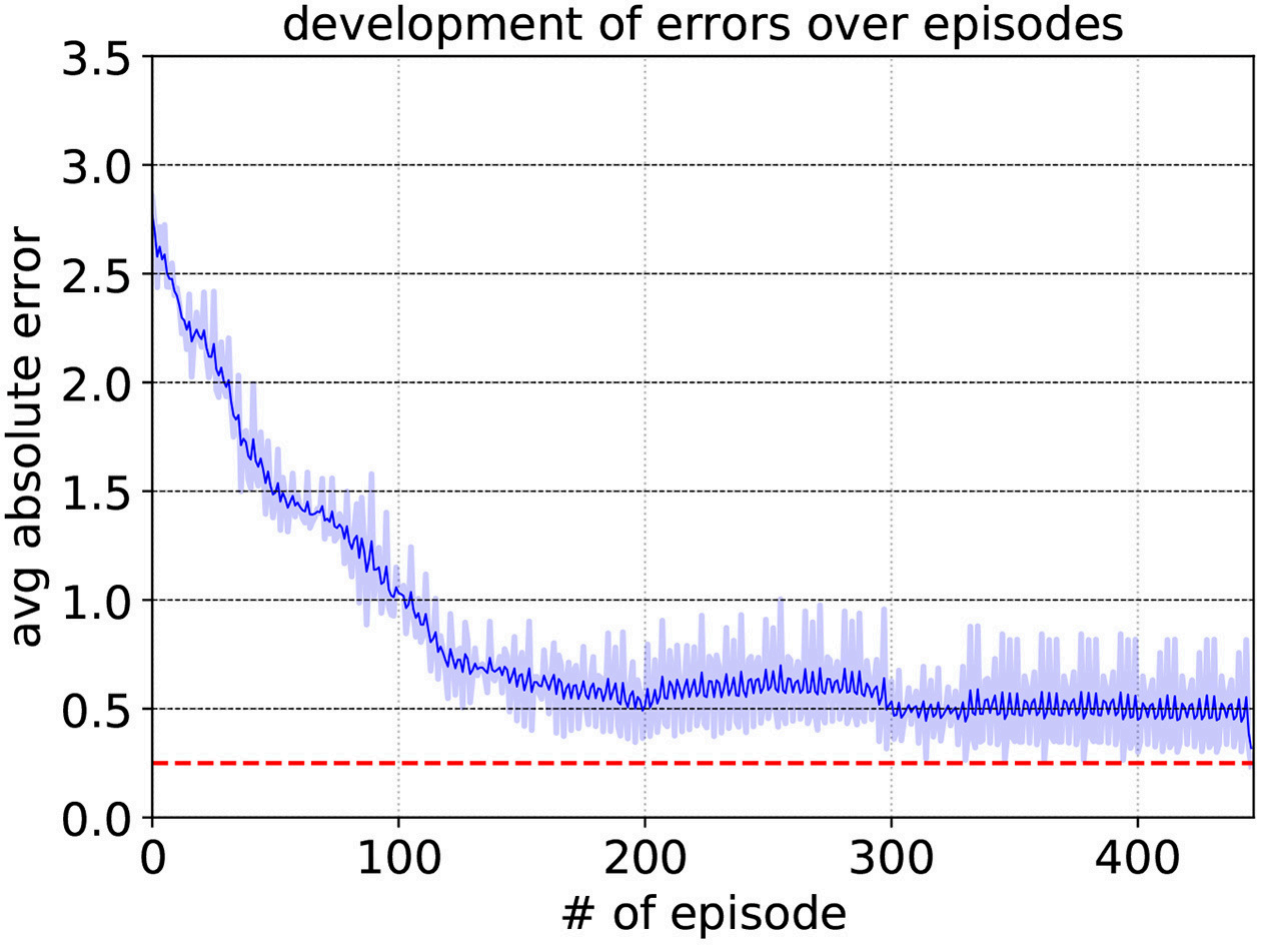
Development of the target neuron's average deviation from *y*_*th,TN*_ for *A*_+_ = 0.1, *A*_−_ = 0.105, η_*max*_ = 0.2, and η_*min*_ = 0.05. The dashed red line represents the maximum deviation of 0.25 that had to be surpassed. The shaded region indicates the average absolute error of each episode. To show the variation trend in a clear way, the solid blue line means the moving mean value of the averaged absolute error over the episode.

Since |*g*_*y*_| = 0.15 results in a desired output angle of approximately 8.6°, an angle close to this value will be interpreted as facing the target. Any other goal vector $\vec{g}$ that does not fall between these two edge cases (−0.15 ≤ *g*_*y*_ ≤ 0.15) means the robot is not facing the target. And consequently, the higher input value |*g*_*y*_| will apparently cause the output to cross the threshold value *y*_*th*_. The learning parameters of the special neurons are set to the same values as for their respective sub-controllers, with the exception of *A*_+_ = 0.1, allowing for overall smaller weight changes. As can be seen in Figure 8, it reaches an average deviation of 0.248 after approximately 340 episodes, where in one episode the edge cases are fed into the SNN five times.

#### 6.2.2. Performance

**Figure 9:**
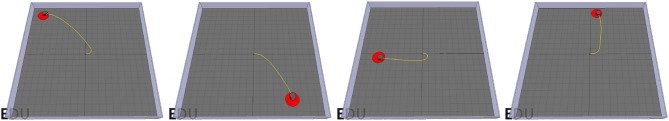
The P3-DX's trajectories controlled by the trained goal-approaching sub-controller for different goal positions and a target radius *r*_*target*_ = 0.3*m*.

The trajectories of the robot controlled by the goal-approaching sub-controller for different initial positions and orientations can be seen in Figure 9. The radius of the target area is set to 0.3 m in all tests. It can be observed that the robot manages to quickly turn toward the goal and reach the target area for all the initial orientations.

**Figure 10:**
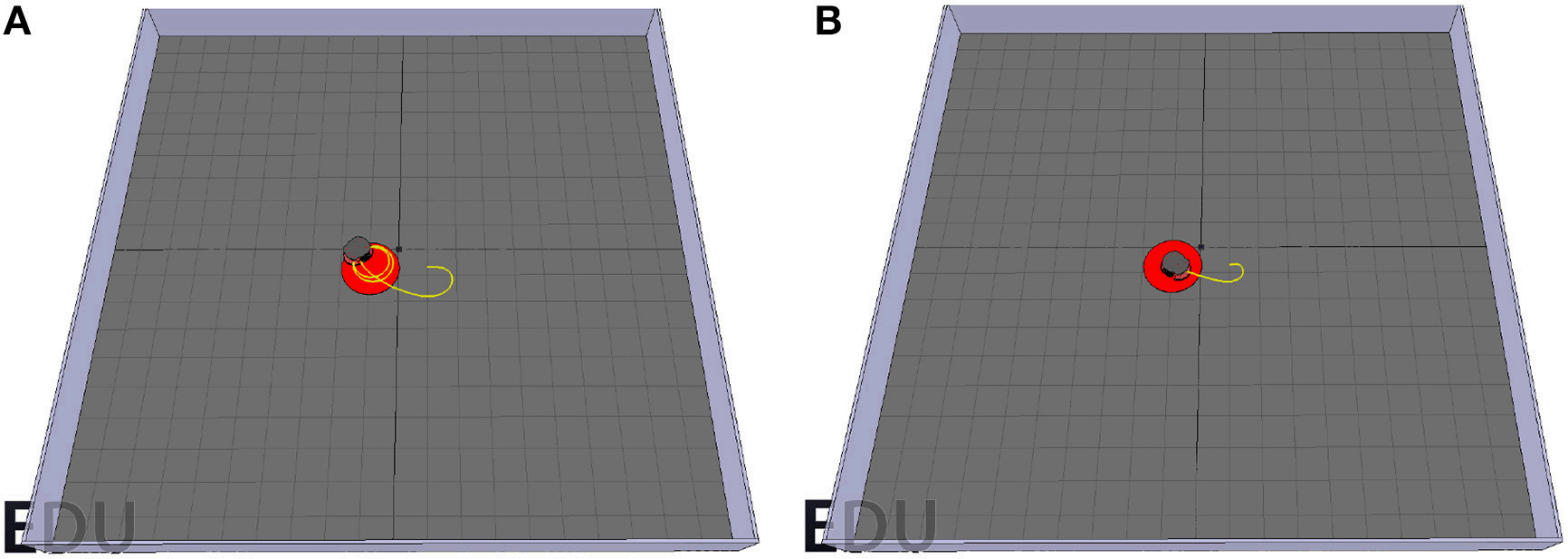
**(A)** The Pioneer robot drives in circles around the target center. **(B)** By reducing the forward velocity according to 23, the pioneer manages to reach the target area.

However, a problem arises while testing. When setting the radius of the target area to a sufficiently small value such as 0.1 m, the Pioneer robot drives toward the target and then, instead of moving closer to it, starts driving around the target area in circles (Figure 10A). This happens because the goal-approaching controller causes the Pioneer robot to turn toward the target, but the consistent average velocity prevents it from moving closer to the target center. However, this could be easily fixed by lowering the mean velocity when getting close to the target center (*d* < 1 m):

(22) $$\begin{array}{l} v_{forward}=\{\begin{array}{ll} v_{init}\times d, & if\;d<1,\\ v_{init}, & else \end{array} \end{array}$$

where *v*_*init*_ is the initial forward velocity when starting the simulation. *d* is the distance between the robot and the target defined in (18). Figure 10B shows the trajectory of the robot with the improvement under the same situation.

### 6.3. Obstacle-Avoiding Sub-controller

#### 6.3.1. Training Details

**Figure 11:**
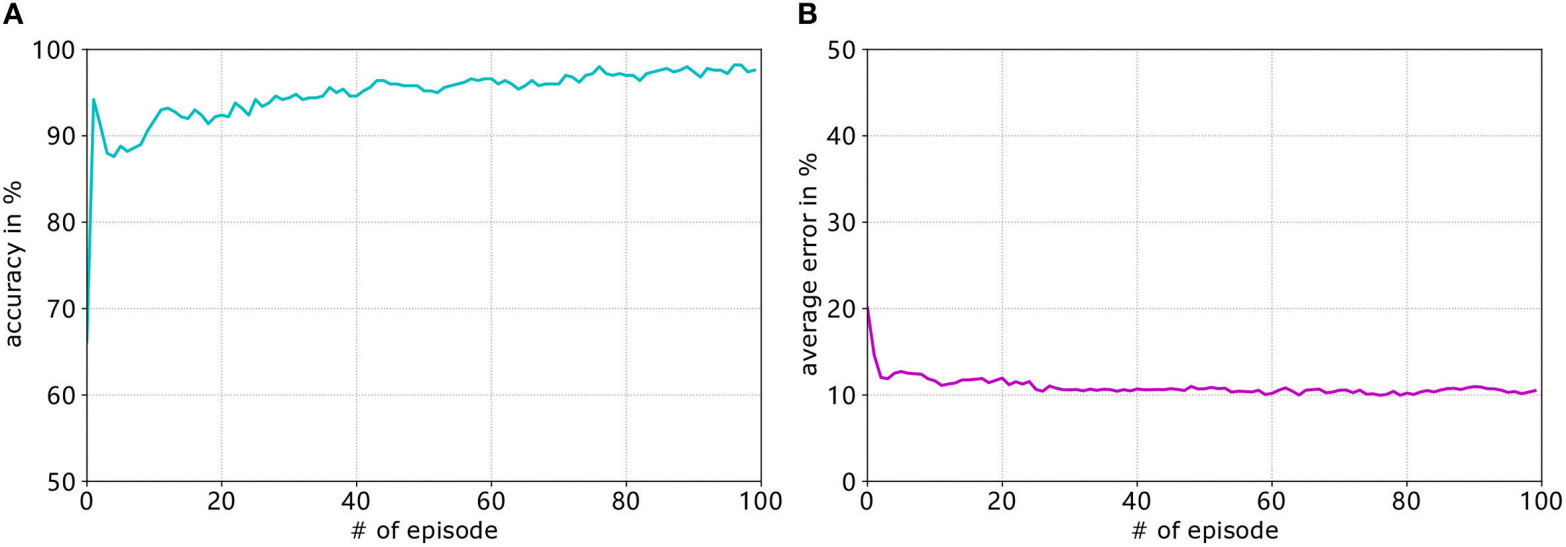
Development of the accuracy (**A**. blue graph) and average error (**B**. purple graph) of the obstacle-avoiding sub-controller over the training episodes for *A*_+_ = 0.4, *A*_−_ = 0.42, η_*max*_ = 0.09, and η_*min*_ = 0.02.

For avoiding obstacles, the learning parameters are set to η_*max*_ = 0.09, η_*min*_ = 0.02, and *A*_+_ = 0.4. The learning process can be seen in Figure 11. Similar to the goal-approaching sub-controller, the accuracy quickly rises to over 90% and continues to increase before it stagnates at around 97.6%. The average error however falls much faster, reaching a value of approximately 10.5% after 30 episodes and stagnating around that rate. The accuracy after the final episode amounts to 97.6%, while the average error rate is 10.5%.

**Figure 12:**
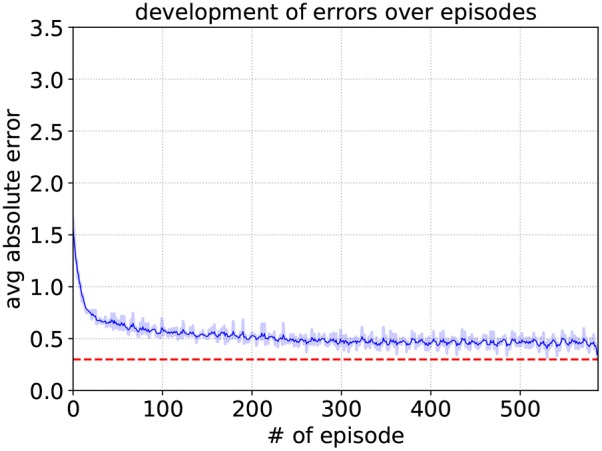
Development of the obstacle neuron's average deviation from *y*_*th,TN*_ for *A*_+_ = 0.1, *A*_−_ = 0.105, η_*max*_ = 0.2, and η_*min*_ = 0.05. The dashed red line represents the maximum deviation of 0.3 that had to be surpassed.

The obstacle neuron undergoes the same training procedure as the target-facing neuron. The development of the average deviation per training episode can be seen in Figure 12. The maximum deviation is set to 0.3 and it is surpassed after approximately 580 episodes. For the obstacle neuron, the edge cases are the six sensor readings from (15), while every other sensor reading is set to 1 (not detecting anything). Since the input neurons exhibit spikes even when not detecting anything, the case where no sensor detects anything is also included in the edge cases. For this case, however, instead of approximating the value *y*_*th,ON*_, it is trained to assume a value lower than *y*_*th,ON*_/2 to make sure the unconditioned firing does not cause the neuron to fire too early, resulting in falsely detecting an obstacle.

#### 6.3.2. Performance

**Figure 13:**
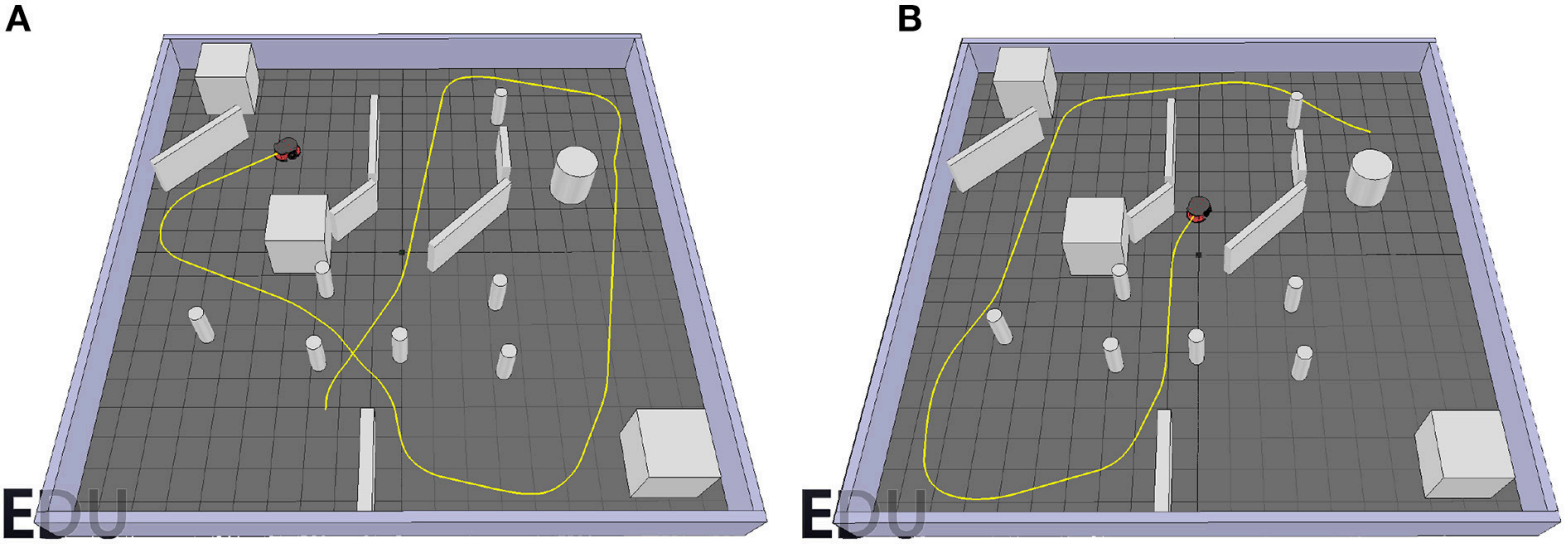
Trajectories of the robot controlled by the obstacle-avoiding sub-controller for two different starting positions. **(A)** First start position. **(B)** Second start position.

**Figure 14:**
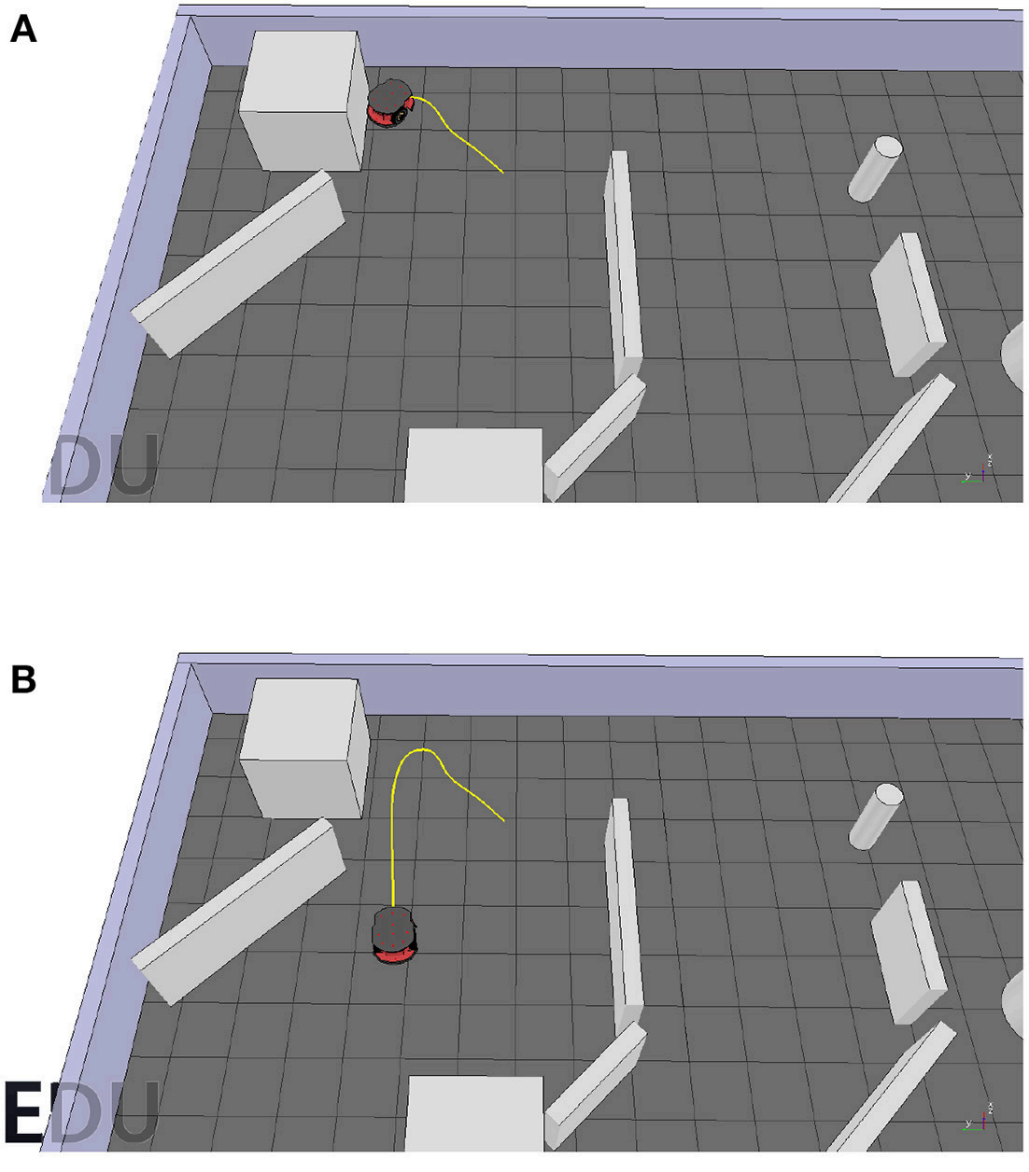
**(A)** The robot fails to avoid the obstacle. **(B)** The robot successfully avoids the obstacle when adjusting its forward velocity according to 24.

Figure 13 shows the robot's trajectory controlled by the obstacle-avoiding sub-controller. It manages to efficiently avoid obstacles while moving around the scene. However, there is one rarely encountered case where it fails. This can also be imputed to the consistent mean velocity of the robot. When directly driving toward a corner, it detects the obstacle too late, such that there is not enough space for taking a turn in any direction, which in turn leads to collision with an obstacle. This is illustrated in Figure 14A. This could be solved however in a similar fashion to the goal-approaching sub-controller by simply relating the default forward velocity to the sensor readings causing a decrease when getting close to an obstacle:

(23) $$\begin{array}{l} v_{forward}=v_{init}\times \overset{6}{\underset{i=1}{\sum }}s_{i}\times \frac{1}{6}, \end{array}$$

This adjustment results in the robot needing much less space for a turning maneuver and therefore allowing for a smoother trajectory when avoiding obstacles. The resulting path solving this problem is shown in Figure 14B.

### 6.4. Overall Performance

**Figure 15:**
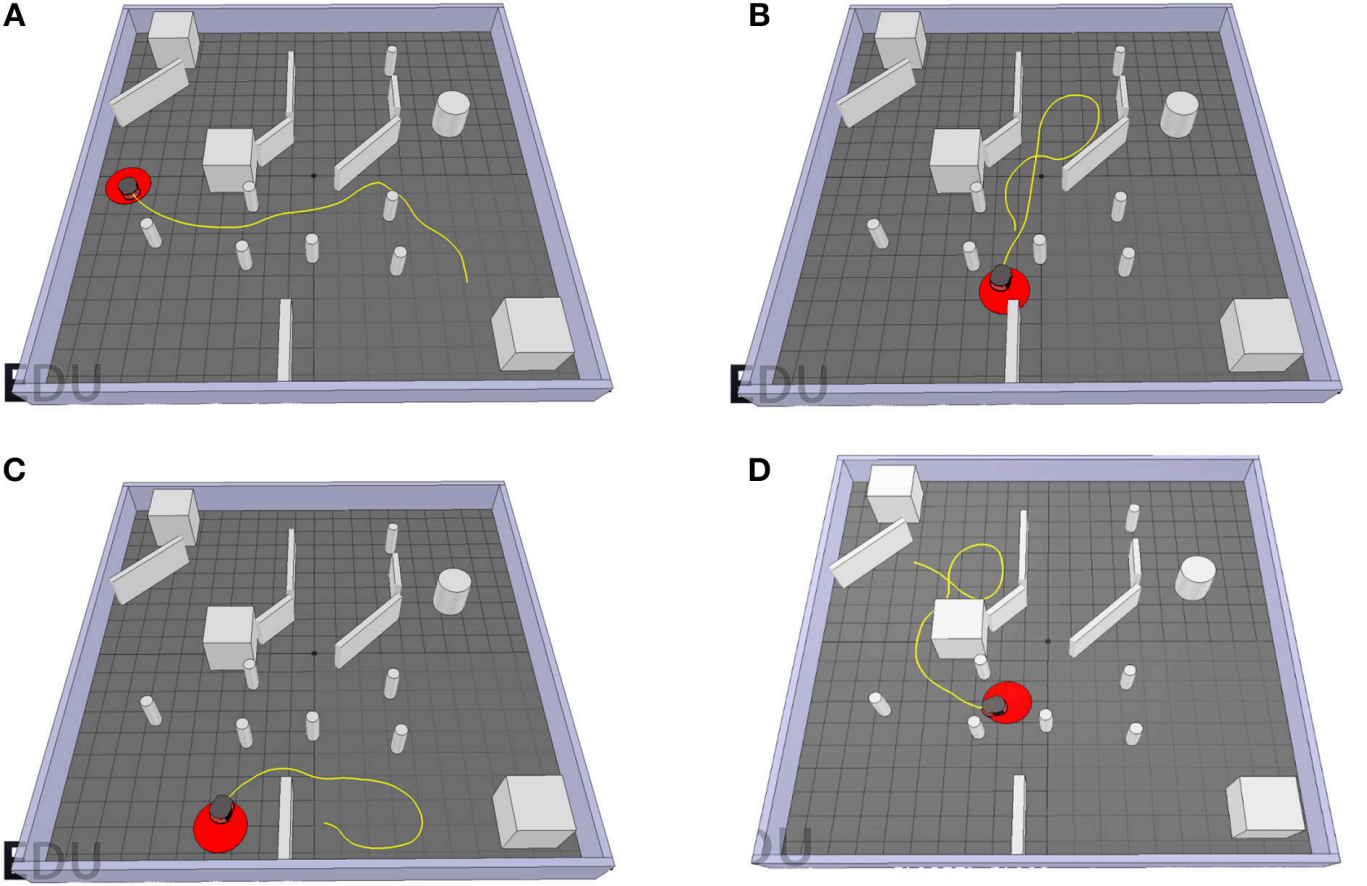
The trajectories of the P3-DX controlled by the TR-Controller for different starting and target positions for a target radius of 0.3*m*. **(A)** The first start position. **(B)** The second start position. **(C)** The third start position. **(D)** The fourth start position.

Combining the two sub-controllers, the target-reaching controller exhibits successful goal approaching behavior while avoiding obstacles in its path. Figure 15 shows the path of the Pioneer robot controlled by the target-reaching controller for different scenarios. In Figure 15A, it maneuvers through a pool of obstacles and quickly reaches the target area. Figure 15B shows the trajectory for the case when there is not enough space to turn to the target. The robot then follows the path until there is enough room for a turning maneuver, and then directly drives toward the target. In Scenario C, the target is behind the Pioneer P3-DX, separated by a wall. In the beginning, the robot tries to make a right turn before detecting a wall. It then moves away from it and turns left to face the target. After moving around the wall behind which the goal is located, i terminates in the target area. Figure 15D shows its behavior when the target is located behind a big concave-shaped obstacle. It avoids it with a left turn and then turns 180° to maneuver around it and finally reach the target. As can be observed, the robot manages to quickly reach the target in every scenario while avoiding all obstacles in its path.

The previous results and performances show that the target-reaching controller as well as the embedded sub-controllers exhibit the desired behavior after being trained with the proposed learning rule. While the accuracy of both sub-controllers rises to a value higher than 95%, the error rates stagnate at approximately 10%. This is because the amount of spike times is limited in each simulated step size (*dt* = 1.0 ms), only allowing for so much precision. This is due to the relatively high step size (*dt* = 1.0 ms) of the simulated SNNs and therefore limited amount of spike times, only allowing for so much precision. This however increases the speed of the simulation, resulting in more updates per second. A higher precision could be achieved by lowering the step size, given the computation speed is not an issue. Moreover, considering that the target output angles provided by the dataset used to train the obstacle-avoiding sub-controller are meant as points of reference and are at a minimum 20° apart from each other, in theory an average error of 10° is appropriate and acceptable for outputting all angles between −90° and +90°.

Even though the controllers and special neurons have to be trained to behave differently, the same learning rule could lead to successful results. Apart from this network being able to be used to train two sub-controllers with multiple outputs on different tasks, it is also shown that the *backpropagation* of the rewards works well and can be easily assigned to each side's synaptic connections, effectively resulting in the training of two different SNNs on a one-dimensional output. This underlines the ability of this proposed approach to effectively train a network on two different outputs at the same time, yielding similar results to a network trained for a single output.

The problems encountered while testing the performance of the sub-controllers can all be accounted for the forward velocity and therefore could be easily solved. However, the target-reaching controller does have its flaws. First, a drawback of this training procedure is the need for a dataset or reference controller providing the SNNs with a desired optimum output value to calculate the reward. In neurology, however, the learning rule only needs some kind of mechanism determining whether the SNNs outputs are too high or too low. The second limitation with the controller can be observed in Figure 15D. Even though the obstacle avoiding and goal approaching work well on their own, the target-reaching controller does not coalesce the different sub-controller's outputs and exclusively either avoids obstacles or approaches the target. This is because the obstacle-avoiding controller is meant to choose the smaller angle between the two output angles for more efficient turning. However, under some circumstance, the larger turning angle leads closer to the target. Therefore, the robot takes some unnecessary steps to reach the final target.

## 7. Discussions and Limitations

This paper presented an approach for fast building an SNN-based controller for performing robotic implementations. Our approach first used a model-based control method to shape a desired behavior of the robot as a dataset and then use it to train an SNN based on supervised learning. We presented a robot navigation task as a case study to demonstrate our proposed approach. Specifically, we have demonstrated that pre-acquired knowledge can be used for training an SNN with R-STDP synapses to achieve desired functions. We have also demonstrated that the reward can be assigned properly to all the synapses in an SNN constructed with hidden layer. Finally, our proposed method has been demonstrated on a simulated robot navigation task. The SNN-based controller can quickly assemble the knowledge from the dataset and exhibit adaptiveness in unknown environment.

It is worth noting that, we do not claim the dataset of our approach can be used for different tasks without modifications. The motivation of our approach is to present an alternative to train SNNs quickly for practical implementations, where we expect that SNN-based controllers can exhibit their advantages on neuromorphic hardware. Our approach also requires a pre-acquired dataset to train the SNNs off-line based on the supervised learning framework. However, this problem is expected to be solved when the network equips memory-like functions to store its knowledge and train itself at the same time or afterwards.

## 8. Conclusion and Future Work

Teaching a brain-inspired spiking neural network in a general and easy way is not simple. We tackled this problem by proposing an end-to-end learning rule based on the supervised R-STDP rule and used it for training two SNNs for an autonomous target-tracking implementation. By simply changing the inputs fed into the network and slightly changing the way that the reward was assigned to the output neurons, two SNNs were trained to learn to exhibit the desired behavior successfully and the robot was able to reach a previously set target area while avoiding obstacles.

Our study not only offers a general-purpose training framework for SNNs with multiple outputs and hidden layers but also indicates that how the reward can be properly back-propagated through them.

Together with this, the basic idea of this learning rule also allows for potentially greatly increasing the energy efficiency of SNNs by making them able to learn with and operate on very few and even single spikes per time window.

For future work, we will transfer our approach on real-life robot tasks, which runs a neuromorphic hardware. Thus, we can evaluate the effectiveness of our approach for fast building an applicable SNN-based controller for mobile robot implementations. Although our study proved to work well for networks with one hidden layer and two output neurons, it has yet to be thoroughly tested on how more output neurons affect training and how it performs when increasing the amount of hidden layers in the network. The insights gained could help to further improve this concept up to the point of creating a general-purpose and easy-to-use spiking neural network design for training and energy-efficient control of autonomous mobile robots.

## Author Contributions

ZB and AK brought up the core concept and architecture of this manuscript. ZB and IB designed the experiments. ZB, IB, ZJ, CC, and KH wrote the paper.

### Conflict of Interest Statement

The authors declare that the research was conducted in the absence of any commercial or financial relationships that could be construed as a potential conflict of interest.

## Appendix

In Tables A1, A2, *syn1* denotes the synapse connecting the input to the hidden layer, *syn2* denotes the synapse connecting the hidden to the output layer.

**Table A1:** Training parameters of the goal-approaching controller.

Goal-approaching	Number of training	100
	Episodes	
	Number of test labeled	500
	Data per episode	
	Syn1 initial weights	
	Inhibitory (20%)	−4 ± 1
	Excitatory (80%)	21.5 ± 3.5
	Syn2 initial weights	
	Inhibitory (20%)	−2.5 ± 0.5
	Excitatory (80%)	4 ± 1
	Max. learning rate	η_*max*_ = 0.2
	Min. learning rate	η_*min*_ = 0.05
	Amplitude of weight change	*A*_+_ = 0.4
	for facilitation	
	Time window size for	τ_+_ = 10*ms*
	faciliation	
	Amplitude of weight change	*A*_−_ = 1.05*A*_+_ = 0.42
	for depression	
	Time window size for	τ_+_ = 10*ms*
	depression	
	Maximum weight	*w*_*max*_ = 50
	Minimum weight	−*w*_*max*_/2 = −25
	Eligibility trace	*c*_1_ = 1/*w*_*max*_ = 0.02
	constants	*c*_2_ = 1
	Maximum output	*y*_*max*_ = 5
Target-facing neuron	Initial weights	15
	Max. learning rate	η_*max*_ = 0.2
	Min. learning rate	η_*min*_ = 0.05
	Amplitude of weight change	*A*_+_ = 0.1
	for facilitation	
	Time window size for	τ_+_ = 10*ms*
	faciliation	
	Amplitude of weight change	*A*_−_ = 1.05*A*_+_ = 0.105
	for depression	
	Time window size for	τ_+_ = 10*ms*
	depression	
	Maximum weight	*w*_*max*_ = 50
	Minimum weight	−*w*_*max*_/2 = −25
	Eligibility trace	*c*_1_ = 1/*w*_*max*_ = 0.02
	constants	*c*_2_ = 1
	Maximum output	*y*_*max*_ = 5

**Table A2:** Training parameters of the obstacle-avoiding controller.

Obstacle-avoidance	Number of training	100
	episodes	
	Number of test labeled	500
	data per episode	
	Syn1 initial weights	
	inhibitory (20%)	−3.125 ± 0.625
	excitatory (80%)	6.25 ± 1.25
	Syn2 initial weights	
	inhibitory (20%)	−0.625 ± 0.625
	excitatory (80%)	3.75 ± 1.25
	Max. learning rate	η_*max*_ = 0.09
	Min. learning rate	η_*min*_ = 0.02
	Amplitude of weight change	*A*_+_ = 0.4
	for facilitation	
	Time window size for	τ_+_ = 10*ms*
	faciliation	
	Amplitude of weight change	*A*_−_ = 1.05*A*_+_ = 0.42
	for depression	
	Time window size for	τ_+_ = 10*ms*
	depression	
	Maximum weight	*w*_*max*_ = 50
	Minimum weight	−*w*_*max*_/2 = −25
	Eligibility trace	*c*_1_ = 1/*w*_*max*_ = 0.02
	constants	*c*_2_ = 1
	Maximum output	*y*_*max*_ = 5
Obstacle neuron	Initial weights	6.5 ± 0.5
	Max. learning rate	η_*max*_ = 0.09
	Min. learning rate	η_*min*_ = 0.02
	Amplitude of weight change	*A*_+_ = 0.1
	for facilitation	
	Time window size for	τ_+_ = 10*ms*
	faciliation	
	Amplitude of weight change	*A*_−_ = 1.05*A*_+_ = 0.105
	for depression	
	Time window size for	τ_+_ = 10*ms*
	depression	
	Maximum weight	*w*_*max*_ = 50
	Minimum weight	−*w*_*max*_/2 = −25
	Eligibility trace	*c*_1_ = 1/*w*_*max*_ = 0.02
	constants	*c*_2_ = 1
	Maximum output	*y*_*max*_ = 5
