# Supplementary material for: Supervised Learning in SNN via Reward-Modulated Spike-Timing-Dependent Plasticity for a Target Reaching Vehicle
Source: Front Neurorobot. 2019 May 3;13:18. doi: 10.3389/fnbot.2019.00018 (PMC6509616; doi:10.3389/fnbot.2019.00018)
Supplement: Supplementary file 1 [file Data_Sheet_1.ZIP › SNN-Controller_final/READ-ME.docx]

**Autonomous Locomotion Control of a Mobile Robot Using Spiking Neural Networks**

**DROPBOX**

**FOLDER:**

- ***Controller:*** contains *trained* controller ready to perform its task
- ***Training:*** contains all files needed to train the Sub-Controller and special neurons
- ***V-Rep-Scenes:*** contains all used V-Rep Scenes (Training Environment OA: No. 0002; Testing GA: No. 0005; Testing OA & whole controller: No. 0004)

**TRAINING THE DIFFERENT (SUB-)CONTROLLER** *(all Codes in “Training” folder)*

**Goal Approaching:**

- Edit file parameters.py to set various training and simulation parameters
- Open File: **RSTDP_simulationLearningGA.py** (to train GA network)
- Open File: **RSTDP_simulationLearningGN.py** (to train special neuron)
- Run Files and set amount of training episodes
- When Code is finished: Specify whether you want to save training results (for error/accuracy plots) and/or accumulated weights
- When satisfied with results:
  - Move txt-files containing trained weights to folder ***“Controller->Weights”***
  - Move txt-files containing error/accuracy to folder ***“Training -> AccErr”***
  - Run python-codes located in ***“Training -> AccErr”*** to create the different error/accuracy plots

**Obstacle Avoidance:**

- See Goal Approaching but replace **RSTDP_simulationLearningGA.py** and **RSTDP_simulationLearningGN.py** by **RSTDP_simulationLearningOA.py** and **RSTDP_simulationLearningON.py**

**EXECUTING THE DIFFERENT (SUB-)CONTROLLER**

1. Start V-Rep and open Scene (depending on what (Sub-)Controller you want to test)
2. Open file: ***Controller ->* controllerSNN_final.py**
3. Start V-Rep Simulation
4. Run Python Code:
   1. Specify (Sub-)Controller you want to test (‘GA’, ‘OA’ or ‘both’)
   2. If necessary (depending on controller), specify connectivity type (for OA; ‘split’ or ‘blackbox’) and/or target radius (for GA; in meter)

**RELEVANT py-FILES LOCATED IN TRAINING FOLDER:**

- **error_plots.py:** contains functions to plot error/accuracy while training
- **parameters.py:** to set various training and simulation parameters
- **RSTDP_networkGA.py:** contains the SNN model for GA
- **RSTDP_networkOA.py:** contains the SNN model for OA
- **RSTDP_simulationLearningGA.py:** file to train SNN for GA
- **RSTDP_simulationLearningGN.py:** file to train SNN for OA
- **RSTDP_simulationLearningOA.py:** file to train special neuron GN/TN
- **RSTDP_simulationLearningON.py:** file to train special neuron ON

**RELEVANT py-FILES LOCATED IN CONTROLLER FOLDER:**

- **parameters.py:** see previous section
- **RSTDP_networkGA.py:** see previous section
- **RSTDP_networkOA.py:** see previous section
- **vrepCode.py:** file containing functions to communicate with V-Rep
- **vrep.py:** by V-Rep; necessary for communication with V-Rep
- **vrepConst.py:** by V-Rep; necessary for communication with V-Rep
- **controllerSNN_final.py:** actual controller controlling the P3-DX

***ATTENTION:*** *the file* ***remoteAPI.dll*** *is tailored for the different OS available (windows 32bit and windows 64bit); if the code does not run (especially the communication with V-Rep) consider replacing it by the file for you OS located in you V-Rep folder in:* ***V-REP_PRO_EDU -> programming -> remoteApiBindings -> lib -> lib -> [your Windows OS]***
